# Age-stratified neurosurgical outcomes for traumatic brain injury in a pediatric neurosurgical cohort in La Paz, Bolivia

**DOI:** 10.1007/s00381-026-07134-x

**Published:** 2026-01-17

**Authors:** Caleigh S. Roach, Jacob J. Shawwa, Connor Nee, Shreyas Chetan, George Dong, Anthony Rios, Jorge Daniel Brun, Jorge David Brun, Victor M. Lu

**Affiliations:** 1 Department of Neurosurgery, University of Miami, Miami, FL USA; 2 Miller School of Medicine, University of Miami, Miami, FL USA; 3 Department of Neurological Surgery, Hospital del Niño “Dr. Ovidio Aliaga Uria”, La Paz, Bolivia

**Keywords:** Neurosurgery, Bolivia, Pediatric neurosurgery, Latin America, Health equity

## Abstract

**Purpose:**

To assess age-stratified differences in neurosurgical outcomes following pediatric traumatic brain injury (TBI) in a resource-limited setting, using World Health Organization-defined developmental categories.

**Methods:**

A retrospective review was conducted of pediatric TBI cases requiring neurosurgery at a tertiary hospital in La Paz, Bolivia (2019–2023). Primary outcomes included mortality and postoperative complications. Secondary outcomes were admission-to-surgery time, 30-day reoperation/readmission rates, and hospital length of stay (LOS).

**Results:**

A total of 165 cases were identified with a median age of 4.7 years (IQR 1.3–8.3). Infants had the highest rates of postoperative complications (44%) and reoperations (28%), significantly greater than older children (*p* < 0.01). Infants experienced longer admission-to-surgery delays (median 3 vs. 1 day, *p* < 0.001) and nearly double the LOS (median 21 vs. 9 days, *p* < 0.001). Children aged 6–16 years more frequently had focal injuries, typically underwent surgery within 1 day, and showed favorable short-term outcomes. Overall, 30‑day mortality was 4% (*n* = 6), with four deaths within 48 h postoperatively. While overall 30-day survival (96%) did not differ by age (log-rank *p* = 0.45), reoperation-free survival varied significantly (*χ*^2^ = 15.3, *p* < 0.01). Overall, 12% required reoperation by 30 days, primarily driven by infants (28% vs. 7% in older children; *p* = 0.02).

**Conclusion:**

Younger age, particularly infancy, was associated with higher surgical complexity, delays in intervention, and increased complications and reoperations, despite similar survival. Age-specific clinical protocols and early resource prioritization are essential to improve pediatric TBI outcomes in resource-limited environments.

## Introduction

Traumatic brain injury (TBI) is a critical global health issue and the leading cause of trauma-related death and disability in the pediatric population [1]. Each year, over 3 million children worldwide experience TBI, and many of them require subsequent neurosurgical intervention [2, 3]. Age is among the strongest predictors of TBI outcomes, with younger children particularly vulnerable to severe injury effects [4, 5]. The anatomy of infants and toddlers, including pliable skulls, open cranial sutures, and immature neck musculature, significantly influences injury mechanisms and clinical course [6]. Studies have shown that moderate-to-severe TBI before age 25 significantly raises mortality risk compared to unaffected peers, with even greater challenges in diagnosis and management for younger individuals [7–12].

The burden of pediatric TBI is disproportionately higher in low- and middle-income countries (LMICs). Greater injury incidence and resource and training limitations contribute to higher pediatric TBI mortality compared to high-income countries [4, 13]. Bolivia, like many other LMICs, faces challenges in pediatric neurotrauma care, including geographic barriers, variability in initial management, and resource constraints in critical care [14–16]. To date, the understanding of pediatric TBI management in Bolivia is largely unknown. This study focuses on pediatric TBI patients treated with neurosurgical interventions at the Hospital del Niño in La Paz, Bolivia, a high-altitude city serving a large catchment area encompassing urban and rural communities. This study aims to evaluate age-dependent trends in neurosurgical TBI outcomes and inform the development of health system interventions to improve pediatric TBI outcomes in low-resource settings.

## Methods

### Study design and setting

A retrospective cohort study was conducted of pediatric TBI patients treated at the principal public tertiary hospital in La Paz, Bolivia. This hospital serves as a referral center for neurosurgical care for La Paz and the surrounding highland provinces. Cases were identified through the Sistema de Información Clínico Estadístico (SICE) electronic service records. Institutional research ethics approval was obtained through the Hospital del Niño research committee prior to data collection.

### Patient inclusion and exclusion criteria

Patients aged 0–16 years who underwent a neurosurgical procedure for TBI between January 2019 and December 2023 were included. TBI was defined as any head injury due to an external force resulting in intracranial pathology requiring neurosurgical attention. Neurosurgical intervention was broadly defined to encompass cranial operations, such as craniotomy or craniectomy for hematoma evacuation or decompression, elevation of depressed skull fractures, burr hole placement for fluid drainage, ventricular shunt procedures for post-traumatic hydrocephalus, and any other operative interventions performed by the neurosurgery team as part of acute TBI management. Patients with non-traumatic intracranial surgeries were excluded. If a patient underwent multiple TBI-related interventions, only the initial admission was considered for primary outcomes to avoid double-counting; however, subsequent related admissions were noted in follow-up data.

### Data collection and classification

Using the SICE electronic health record, demographic data (age in years/months, sex, home province), injury details (mechanism of injury, transfer status), radiological findings, surgical details, hospital course, and 30-day outcomes were collected. Mechanism of injury (MOI) was categorized as falls, motor vehicle collision (MVC), sports-related, or unspecified trauma. Neurosurgical interventions were classified by type and approach/laterality. The admission-to-surgery time was calculated as the interval from hospital admission to the start of the first neurosurgical procedure, in days. Operative duration was recorded from the start of incision to closure (in hours). Intraoperative complications and postoperative complications during hospitalization were documented. The postoperative length of stay (LOS) in hospital (days from surgery to discharge) and total LOS were recorded. Any return to the operating room during the same admission and any readmission to the emergency department within 30 days requiring neurosurgical intervention was documented. The discharge outcome was recorded as alive or in-hospital death. Overall survival was defined as in-hospital or post-discharge death occurring within 30 days of initial operation. Invasive intracranial pressure (ICP) monitoring was not available at the study institution during the study period.

### Age group stratification

For subgroup comparison, patients were stratified into age groups based on the World Health Organization (WHO)-recommended tiered set of early life age groups. The age categories used were as follows: newborn (birth to < 1 month), infant (1 to < 12 months), toddler (1 to < 2 years), early childhood (2 to < 6 years), middle childhood (6 to < 11 years), early adolescence (11 to < 16 years) [17, 18].

### Statistical analysis

The primary outcomes were in-hospital mortality and the incidence of major postoperative complications across different age groups. Secondary outcomes included operative duration, admission-to-surgery interval, total hospital LOS, reoperation rate, and 30-day post-discharge return for surgery, stratified by age group. Continuous variables were presented as medians with interquartile ranges (IQR) and compared using non-parametric tests (Mann-Whitney U or Kruskal-Wallis ANOVA for multiple groups). Categorical variables were compared using Chi-square or Fisher’s exact tests as appropriate. Categorical outcomes were modeled with univariate logistic regression and continuous outcomes with simple linear regression. For logistic models, results were expressed as odds ratios with 95% confidence intervals, pseudo-*R*^2^, and the area under the ROC curve (AUC). Linear models reported the unstandardized β-coefficient with 95% confidence limits and the adjusted *R*^2^. Kaplan-Meier survival analysis was performed to assess the 30-day time-to-event for two primary clinical endpoints: overall survival and reoperation-free survival. Patients were stratified a priori into the six developmental categories based on age, utilizing middle childhood as the reference category. A two-sample log-rank test compared overall survival with reoperation-free survival, while multigroup log-rank tests assessed age-stratified differences for reoperation-free survival. Probabilities were expressed on a percent scale. Hazard ratios (HRs) were estimated with Cox proportional-hazards regression. Age-adjusted models were fitted for overall survival and reoperation-free survival. Age categories exhibiting zero events were handled with a mild ridge penalty (λ = 0.1) to ensure convergence. Model results are reported as HR = e^β with 95% confidence intervals and two-sided *p*-values. Statistical significance was set at a two-tailed *p* < 0.05, and all analyses were conducted in Python version 3.11.

## Results

### Cohort characteristics

**Fig. 1:**
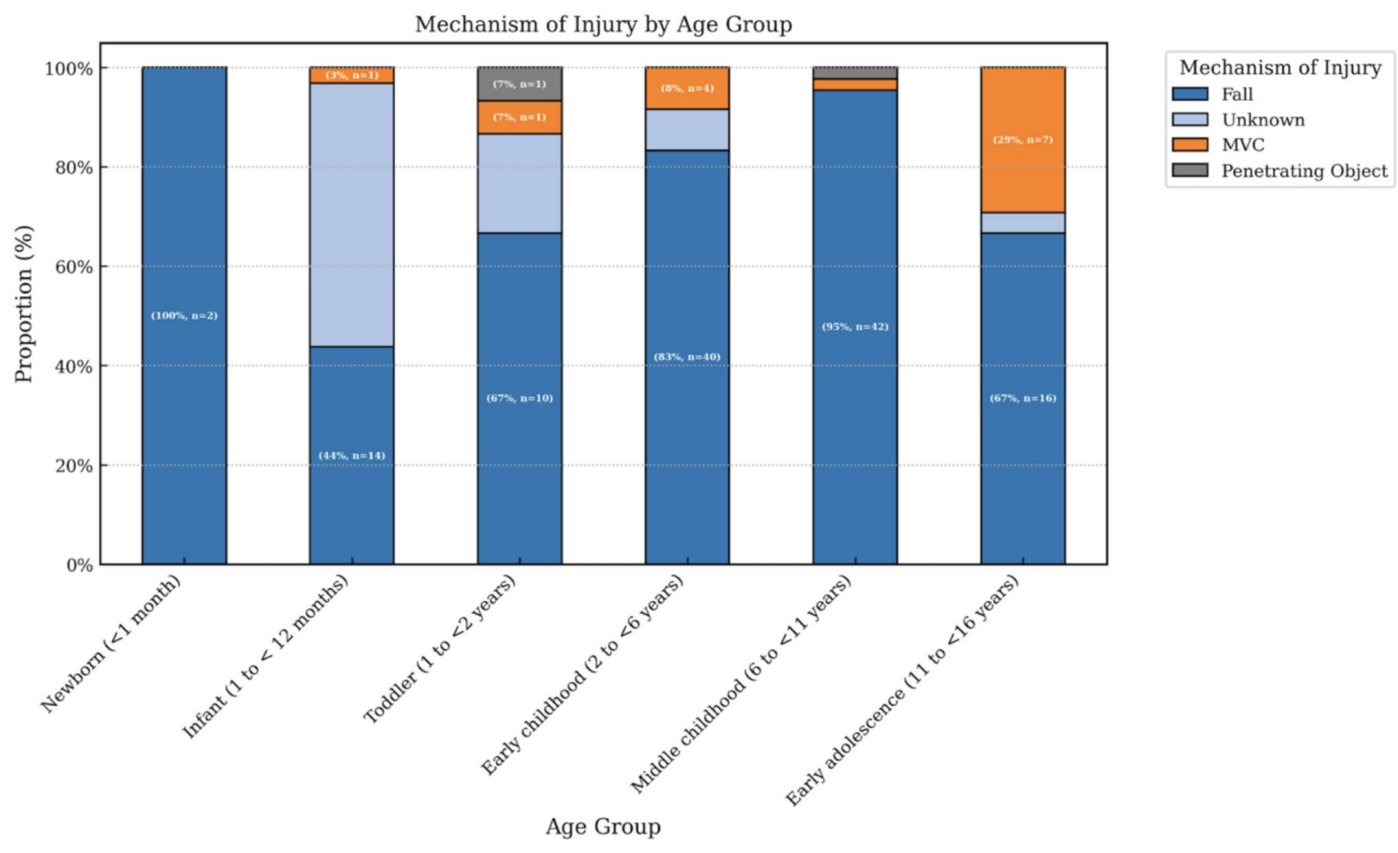
Presenting mechanism of injury stratified by patient age. Mechanism of injury stratified by age group for pediatric patients undergoing neurosurgical intervention. Falls were the predominant cause of injury across all age categories, with motor vehicle collisions (MVC) increasing notably in early adolescence. Percentages and case counts (*n*) are indicated within each bar segment

Between 2019 and 2023, 165 children underwent neurosurgical intervention for treatment following TBI. Table 1 presents the cohort characteristics stratified by age group. The cohort was split between males and females nearly evenly (51% vs 49%, respectively), with a median age of 4.7 years (IQR, 1.3–8.3). Early childhood (3–5 years) was the largest group (29%), followed by middle childhood (27%), infants (20%), and early adolescence (15%). Only two patients were newborns (1.2%). Mechanism of injury varied significantly with age (*p* < 0.001). Falls were the dominant cause overall (75%) and especially common in early childhood (83%). Motor vehicle collisions (MVCs) accounted for 8.5% of cases (*n* = 14), occurring primarily in the early adolescent group (29%) (Fig. 1).

**Table 1:** Demographic and clinical characteristics of pediatric patients undergoing neurosurgical intervention, stratified by age group

Age groups	Cohort (***N*** = 165)	Newborn % (*n*)	Infant % (*n*)	Toddler % (*n*)	Early childhood % (*n*)	Middle childhood % (*n*)	Early adolescence % (*n*)
*Study cohort*	**-**	1.2 (2)	19.4 (32)	9.1 (15)	29.1 (48)	26.7 (44)	14.5 (24)
*Demographics*							
Age	4.7y (1.3–8.3)	21.5d (20.3- 22.8)	6.1 m (3.7–7.5)	15.5 m (13.1–17.6)	4.2y (2.9–4.9)	7.7y (6.6–9.2)	12.4y (11.7–13.4)
Female	(*n* = 81)	1 (50.0)	17 (53.1)	7 (46.7)	27 (56.3)	22 (50.0)	7 (29.2)
Male	(*n* = 84)	1 (50.0)	15 (46.9)	8 (53.3)	21 (43.8)	22 (50.0)	17 (70.8)
*Mechanism of injury*							
Fall	(*n = *124)	2 (100)	14 (43.8)	10 (66.7)	40 (83.3)	42 (95.5)	16 (66.7)
MVC	(*n* = 14)	0 (0)	1 (3.1)	1 (6.7)	4 (8.4)	1 (2.3)	7 (29.2)
Penetrating object	(*n* = 2)	0 (0)	0 (0)	1 (6.7)	0 (0)	1 (2.3)	0 (0)
Other/unknown	(*n* = 25)	0 (0)	17 (53.1)	3 (20.0)	4 (8.4)	0 (0)	1 (4.2)
*Presenting diagnosis*							
Epidural hematoma	(*n* = 102)	1 (50.0)	5 (15.6)	8 (53.3)	35 (72.9)	35 (79.5)	18 (75.0)
Subdural hygroma	(*n* = 20)	0 (0)	14 (43.8)	2 (13.3)	2 (4.2)	1 (2.3)	1 (4.2)
Depressed skull fracture	(*n* = 19)	1 (50.0)	4 (12.5)	3 (20.0)	4 (8.4)	6 (13.6)	1 (4.2)
Subdural hematoma	(*n* = 9)	0 (0)	5 (15.6)	0 (0)	2 (4.2)	1 (2.3)	1 (4.2)
Intracerebral hemorrhage	(*n* = 8)	0 (0)	1 (3.1)	1 (6.7)	4 (8.4)	0 (0)	2 (8.3)
Subgaleal hematoma	(*n* = 2)	0 (00	0 (0)	0 (0)	0 (0)	1 (2.3)	1 (4.2)
Other	(*n* = 5)	0 (0)	3 (9.4)	1 (6.7)	1 (2.1)	0 (0)	0 (0)
*Brain region*							
Parietal	(*n* = 65)	1 (50.0)	10 (31.3)	6 (40.0)	21 (43.8)	17 (38.6)	10 (41.7)
Frontal	(*n* = 18)	0 (0)	5 (15.6)	0 (0)	6 (12.5)	6 (13.6)	1 (4.2)
Frontoparietal	(*n* = 10)	0 (0)	1 (3.1)	3 (20.0)	3 (6.3)	2 (4.5)	1 (4.2)
Suboccipital	(*n* = 10)	0 (0)	0 (0)	1 (6.7)	3 (6.3)	4 (9.1)	2 (8.3)
Temporal	(*n* = 9)	0 (0)	2 (6.3)	0 (0)	3 (6.3)	4 (9.1)	0 (0)
Temporoparietal	(*n* = 4)	0 (0)	0 (0)	0 (0)	1 (2.1)	1 (2.3)	2 (8.3)
Other/unknown	(*n* = 17)	0 (0)	0 (0)	4 (26.7)	3 (6.3)	5 (11.4)	4 (16.7)
*Laterality*							
Right	(*n* = 78)	2 (100)	12 (37.5)	9 (60.0)	20 (41.7)	23 (52.3)	12 (50.0)
Left	(*n* = 67)	0 (0)	9 (28.1)	4 (26.7)	25 (52.1)	19 (43.2)	10 (41.7)
Bilateral	(*n* = 16)	0 (0)	11 (34.4)	1 (6.7)	2 (4.2)	0 (0)	2 (8.3)
Midline	(*n* = 4)	0 (0)	0 (0)	1 ((6.7)	1 (2.1)	2 (4.5)	0 (0)
*Neurosurgical procedure*							
Craniotomy	(*n *= 136)	1 (50.0)	24 (75.0)	12 (60.0)	40 (83.3)	39 (88.6)	20 (83.3)
Craniectomy	(*n *= 8)	0 (0)	1 (3.1)	1 (6.7)	3 (6.3)	2 (4.5)	1 (4.2)
Burr hole	(*n* = 8)	1 (50.0)	4 (12.5)	0 (0)	1 (2.1)	1 (2.3)	1 (4.2)
Fracture elevation	(*n *= 6)	0 (0)	0 (0)	1 (6.7)	3 (6.3)	1 (2.3)	1 (4.2)
Other procedure	(*n* = 7)	0 (0)	3 (9.4)	1 (6.7)	1 (2.1)	1 (2.3)	1 (4.2)
*Operative course*							
Time to surgery (days)	1.0 (1.0–3.0)	3.0 (2.0–4.0)	3.0 (1.0–7.0)	1.0 (0.0–1.0)	1.0 (0.0–3.0)	1.0 (1.0–2.0)	1.0 (1.0–2.0)
Operative duration (hours)	1.5 (1.0–2.0)	1 (0.75–1.3)	1 (1.0–1.0)	1.5 (1.5–1.9)	1.5 (1.0–2.0)	1.5 (1.0–2.0)	1.6 (1.3–2.0)
Intraoperative complication	(*n* = 5)	0 (0)	1 (3.1)	2 (13.3)	1 (2.1)	1 (2.3)	0 (0)
*Postoperative course*							
Postoperative complication	(*n* = 33)	0 (0)	14 (43.8)	2 (13.3)	6 (12.5)	2 (4.5)	5 (20.8)
Infection	(*n* = 10)	0 (0)	5 (15.6)	0 (0)	3 (6.3)	1 (2.3)	1 (4.2)
CSF leak	(*n* = 3)	0 (0)	2 (6.3)	0 (0)	0 (0)	0 (0)	1 (4.2)
Postoperative imaging	(*n* = 52)	0 (0)	12 (37.5)	3 (20.0)	10 (20.8)	9 (20.5)	8 (33.3)
Postoperative LOS (days)	8.0 (6.0–13.0)	5.5 (4.3–6.8)	14.5 (7.8–20.3)	9 (6.5–13.5)	8 (6–10.3)	7.0 (5.0–10)	8 (6.14.3)
Total LOS (days)	10.0 (7.0–16.0)	8.5 (8.3–8.8)	21.0 (10.8–29.3)	10.0 (7.5–15.5)	9.0 (7.0–16.0)	9.0 (6.8–13.0)	9.5 (8.0–15.0)
*Outcomes*							
Return to OR during stay	(*n* = 18)	0 (0)	9 (28.1)	2 (13.3)	1 (2.1)	3 (6.8)	3 (12.5)
Length of follow-up (days)	42.0 (19.3–136.8)	251 (251–251)	71 (33–311)	74 (30–165)	47 (18–163)	31 (18–55)	27 (19–90)
Readmission with reoperation	(*n* = 5)	0 (0)	4 (12.5)	1 (6.7)	0 (0)	0 (0)	0 (0)
30-day return to OR	(*n* = 19)	0 (0)	10 (31.3)	3 (20.0)	0 (0)	3 (6.8)	3 (12.5)
30-day mortality	(*n* = 6)	0 (0)	1 (3.1)	0 (0)	1 (2.1)	3 (6.8)	1 (4.2)
30-day reoperation-free survival	(*n* = 140)	2 (100)	21 (65.6)	12 (80.0)	47 (97.9)	39 (88.6)	20 (83.3)

*MVC*, motor vehicle crash, *CSF*, cerebral spinal fluid; *LOS*, length of stay, *OR*, operating room

The table summarizes patient demographics, mechanisms of injury, presenting diagnoses, anatomical injury localization, surgical procedures, operative course details, postoperative complications, length of hospital stay (LOS), and outcomes including mortality and reoperation rates across defined developmental categories. Data are expressed as counts (*n*), percentages (%), or medians with interquartile ranges (IQR).

### Injury patterns

**Fig. 2:**
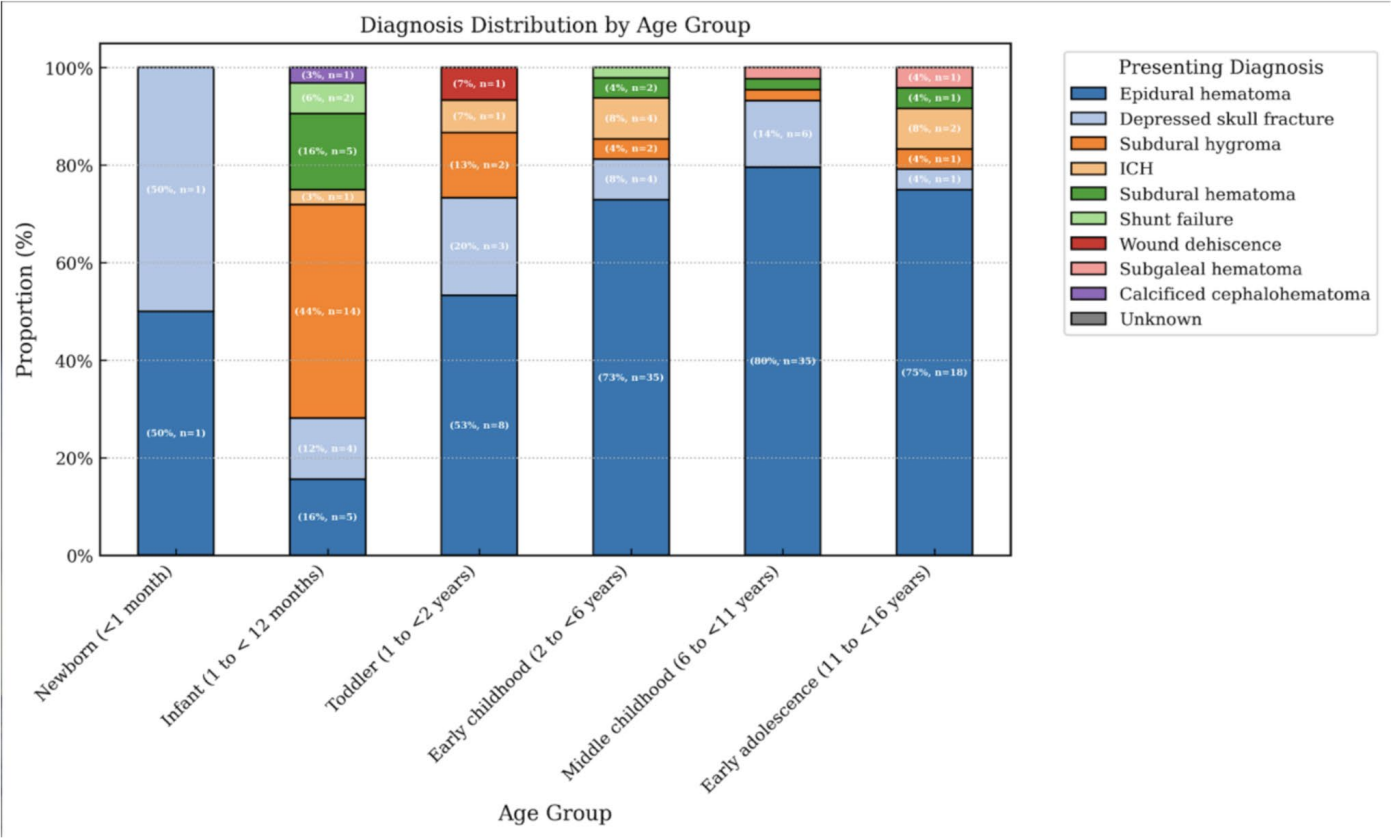
Presenting radiographic injury stratified by patient age. Distribution of presenting diagnoses stratified by age group among pediatric neurosurgical patients. Epidural hematoma (EDH) predominated in older age groups, while subdural hygroma and subdural hematoma were notably prevalent among infants. Case counts (*n*) and percentages are indicated within each segment

Across the cohort, epidural hematoma (EDH) was the single most common diagnosis (62%, *n* = 102), followed by subdural hygromas (12%) and depressed skull fractures (12%). EDH was most prevalent in the middle childhood group (80%) and early adolescents (73%). In contrast, infants rarely had EDH (16%) and instead exhibited a higher incidence of subdural pathologies (60%), including 14 infants (44%) with subdural hygroma and five infants (16%) with subdural hematomas. Depressed skull fractures were observed in each age group, though they were least seen in the age extremes, occurring only once in newborns and once in early adolescents (median age 2.6 y). Intracerebral hemorrhage (ICH) was less common (5%, *n* = 8) and mainly occurred in patients older than 2 years old (75%, *n* = 6). The presenting injury most prevalently involved the parietal region (39%, *n* = 65), followed by the frontal region (11%, *n* = 18). Lateralization of injuries showed that right-sided cranial procedures slightly outnumbered left-sided (47% vs 41%). Only 12% of cases had midline or diffuse involvement requiring a bilateral approach, with infants having the highest rate of bilateral cranial surgeries (*n* = 11, 34%). Infants accounted for 69% of bilateral procedures, and such injuries were significantly associated with this group (*p* < 0.001). Figure 2 illustrates the distribution of injury types across age groups, demonstrating the shift from predominantly subdural fluid collections in infants to EDH in older children.

### Neurosurgical intervention

A range of neurosurgical procedures was performed. Craniotomy for evacuation of hematoma was the most common procedure (82%, *n* = 136), followed by craniectomy for decompression (5%, *n* = 8) and burr hole placement (5%, *n* = 8) for subdural fluid evacuation or external ventricular drain insertion. Burr hole placement was predominantly performed in newborns and infants (*n* = 5, 63%) and significantly associated with both groups (*p* = 0.034 and *p* = 0.038, respectively). Elevation of depressed skull fractures occurred in only 4% of patients (*n* = 6).

### Operative course

The median admission-to-surgery time for the cohort was 1 day (IQR 1–3 days). However, this metric varied significantly by age (*p* < 0.01). Infants experienced the longest delays from admission to neurosurgery (3, IQR 1–7), compared to a median of 1 day for all other age groups. In fact, 50% (16) of infants waited greater than 48 h for surgery, whereas nearly 80% of children over 1 year were in surgery within 24 h of admission. Operative durations differed by procedure and, interestingly, also showed an age association. The median operative time was 1.5 h (± 0.5) for the whole cohort. Infants’ surgeries were significantly shorter on average (median = 1 h) than those for older children (median = 2 h, *p* = 0.04). Intraoperative adverse events were rare (3.0%, *n* = 5) and not significantly different between ages.

### Postoperative course

Postoperatively, all patients were managed in a mixed pediatric/neurosurgery intensive care unit (ICU) or high-dependency unit. The rate of postoperative complications during hospitalization was 20% overall (*n* = 33), including ten cases of postoperative infection and three cases of CSF leaks. However, this rate was highly age-dependent (*p* < 0.001). Infants exhibited the highest postoperative complication rate (44% vs. 19% in older children, *p* < 0.01) and reoperation rate (28% vs. 5.2%, *p* < 0.001). The median total LOS for the cohort was 10 days (IQR 7–16). Infants experienced a markedly longer median LOS at 21 days (IQR 11–29), more than double that of any other group. For early childhood, middle childhood, and early adolescence, median LOS was 9–10 days, and toddlers had a median of 10 days. LOS correlated with age and complication burden, and the difference in LOS between age groups was statistically significant (*p* < 0.001).

### Reoperation and readmission

**Fig. 3:**
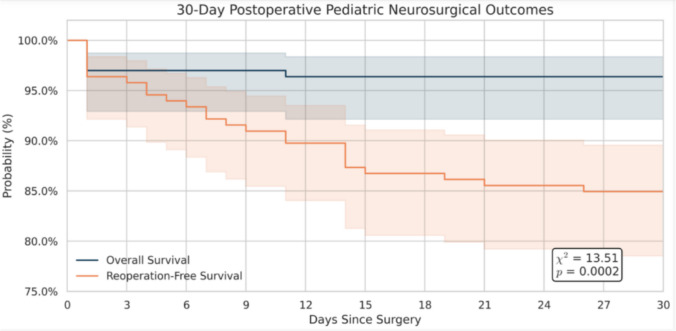
Kaplan-Meier curve of 30-day postoperative survival and reoperation-free survival. Kaplan-Meier curves depicting 30-day postoperative overall survival and reoperation-free survival for pediatric neurosurgical patients. Overall survival remained high throughout the postoperative period, while reoperation-free survival significantly decreased, highlighting early postoperative complications and reoperations (log-rank test: *χ*^2^ = 13.51, *p* = 0.0002). Shaded regions represent 95% confidence intervals

**Fig. 4:**
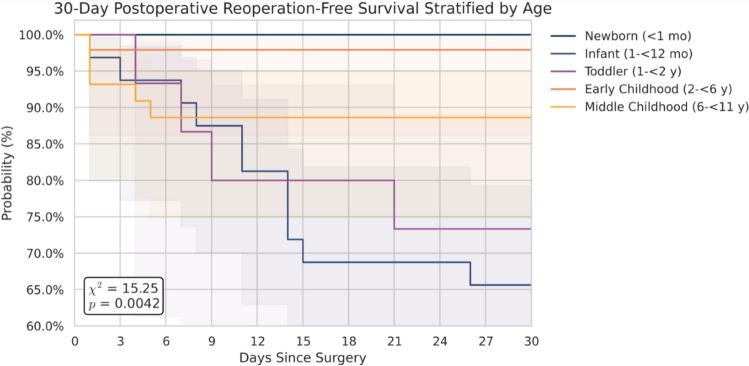
Kaplan-Meier curve of 30-day postoperative reoperation-free survival stratified by age. Kaplan-Meier curves illustrating 30-day postoperative reoperation-free survival stratified by pediatric age groups. The log-rank test represents a global comparison across all groups (*χ*^2^ = 15.25, *p* = 0.0042); individual pairwise differences were not statistically significant. Shaded regions represent 95% confidence intervals

The in-hospital reoperation rate was 11% (*n* = 18) for the cohort, with a significant age-associated difference (*p* = 0.02). Infants experienced the highest in-hospital reoperation rate at 28% (*n* = 9), compared with an incidence of only 7% across the remainder of the cohort. Following the initial hospital stay, five patients required readmission with subsequent reoperation—four of whom were infants (13%) and one a toddler (6.7%). No patient older than 2 years required unplanned readmission and reoperation. Overall, the 30-day incidence of reoperation was 12% (*n* = 19), with four of these patients undergoing a third operation (Fig. 3). Kaplan-Meier analysis demonstrated an early declining trend in reoperation-free survival among infants relative to older age groups, with most events occurring within the first 15 postoperative days (Fig. 4). These findings parallel the higher complication rates and longer hospital stays observed among infants in the cohort.

### Survival

Overall mortality was 4% (*n* = 6), including one infant (3%), one early-childhood (2%), three middle-childhood (7%), and one early-adolescent (4%) death. Four deaths occurred within 48 h of admission due to uncontrollable intracranial hypertension or intraoperative cardiac arrest, and two occurred at 11 and 14 days following prolonged hospitalizations requiring PEG-tube feedings. Age showed a statistically detectable influence on both overall survival (OS) and reoperation-free survival (RFS) when evaluated globally. However, when stratified by age category, 30-day postoperative survival (96%) did not differ significantly between groups (log-rank *p* = 0.45). For RFS, a global log-rank test indicated that survival functions were not identical across all age groups (*χ*^2^ = 15.3, *p* < 0.01), suggesting heterogeneity in postoperative course. Post-hoc pairwise comparisons, however, did not identify a single group responsible for the difference; thus, while infants trended toward lower reoperation-free survival, no individual age category differed significantly from the others. When these age categories were entered into a Cox proportional-hazards model, none of the individual coefficients reached conventional significance (Table 2). Sparse events and small subgroup sizes limited precision. Overall, these findings suggest that while age collectively influences survival patterns, the study is underpowered to determine which specific pediatric strata differ meaningfully from adolescent outcomes after adjustment.

**Table 2:** Patient distribution and unadjusted 30-day outcomes by age group

Age groups	*N*	Deaths *n* (%)	Reoperations *n* (%)	30 d overall survival (95% CI)	30 d reoperation-free survival (95% CI)
Newborn	2	0 (0)	0 (0)	1.00 (-)	1.00 (-)
Infant	32	1 (3.1)	10 (31.3)	0.97 (0.88–1.00)	0.86 (0.78–0.95)
Toddler	15	0 (0)	3 (20.0)	1.00 (-)	0.95 (-)
Early childhood	48	1 (2.1)	0 (0)	0.98 (0.92–1.00)	0.90 (0.82–0.96)
Middle childhood	44	3 (6.8)	3 (6.8)	0.93 (0.82–0.98)	0.83 (0.73–0.91)
Early adolescence	24	1 (4.2)	3 (12.5)	0.96 (0.85–0.99)	0.85 (0.73–0.93)
*Total*	165	6 (3.6)	19 (11.5)	0.96 (0.92–0.99)	0.85 (0.80–0.90)

Patient distribution and unadjusted 30-day outcomes stratified by age group. The table summarizes mortality, reoperation rates, overall survival, and reoperation-free survival at 30 days postoperatively, with corresponding 95% confidence intervals (CI) for each pediatric age category.

## Discussion

In this single-institution retrospective cohort from Bolivia, age at the time of pediatric TBI was strongly associated with differences in injury patterns, management course, and short-term outcomes. Infants and toddlers experienced the highest rates of post-operative complications, reoperations, and prolonged hospital stays, despite having comparable in-hospital survival to older children. Whereas infants had higher incidences of subdural hygroma and diffuse brain injuries, early and middle adolescent children more frequently sustained focal injuries, such as EDH, that could be addressed promptly with favorable outcomes. These age-related differences were especially pronounced in this low-resource setting, reflecting how physiological vulnerability with infrastructural limitations. This study aimed to evaluate these differences and identify actionable targets for age-stratified triage and care allocation in pediatric TBI management.

The association between younger age and worse clinical outcomes aligns with prior research identifying infancy and early childhood as high-risk periods for TBI-related morbidity. Araki et al. reported that children under 4 years of age have elevated trauma mortality, partly due to the diffuse nature of injuries at that age [1]. Although mortality in our study was low (3.7%) and did not significantly differ by age, nearly half of the infants experienced post-operative complications, echoing findings from Bressan et al., who noted higher rates of seizures, hydrocephalus, and cerebral edema in younger patients [3]. The high reoperation rate observed among infants likely reflects the need for staged or delayed interventions for subdural hygromas and hydrocephalus. A tertiary center in Argentina similarly reported frequent multi-stage procedures in infant TBI patients [18]. Kaplan-Meier analysis suggested a trend toward lower reoperation-free survival among infants over the 30-day postoperative period, with most reoperations occurring within 2 weeks. These care outcomes highlight the importance of vigilant monitoring and early follow-up in this vulnerable population. Additionally, the increased postoperative vulnerability of these younger patients is further emphasized by the infants’ increased rates of return to OR within 30 days, return to OR within stay, postoperative complications, and postoperative length of stay in comparison to all other age groups within this study.

Building on these outcome differences, the injury patterns observed were also highly age dependent. EDH predominated in older children and was typically associated with falls, mirroring the statistical findings from global studies [1, 8]. Our surgical cohort showed a particularly high EDH rate (60%) due to selection criteria. In contrast, EDH was rare in infants, who more often presented with subdural hygromas or diffuse cerebral swelling. Some of these patterns suggest abusive head trauma (AHT), where bridging vein rupture and subdural hemorrhage are common [19]. The high incidence of subdural hygromas (59%) may also reflect CSF leakage post-trauma, considered part of the AHT spectrum [19]. Although we did not formally classify AHT, the injury distribution aligns with patterns emphasized by Bressan et al. and others [1, 13], highlighting the importance of screening in this vulnerable age group.

Delays in care access likely contributed to the disparity in complication rates across age groups. In our setting, infants had longer admission-to-surgery intervals, which may reflect diagnostic uncertainty, constrained ICU availability, or conservative initial management. Studies from other LMICs support this concern: in Cambodia, injury-to-hospital delays over 4 h worsened neurologic outcomes [21], while Gupta et al. introduced the concept of the “third delay,” delayed definitive care after hospital arrival, which significantly increased mortality in Indian TBI patients [22]. These findings underscore the importance of expediting neurosurgical triage for infants. In Bolivia, strategies such as telemedicine-based consultations and prioritized transfer protocols could help minimize in-hospital delays and mitigate the risk of secondary injury in the youngest patients [23]. The lack of intracranial pressure (ICP) monitoring capacity highlights the infrastructural challenges faced in Bolivian neurosurgical care. In many LMICs, the absence of monitoring equipment and trained personnel means reliance on clinical examination and imaging alone to detect intracranial hypertension. This gap hampers early detection and management of secondary brain injury, especially among infants, where neurological signs can be subtle. Therefore, expanding access to ICP monitoring and neurocritical care resources should be a priority for capacity-building efforts in low-resource neurosurgical centers [24, 25].

The findings in this study highlight the need for continued investigation into pediatric neurosurgical outcomes and age-specific TBI management in LMICs. Ongoing analysis should inform more tailored clinical strategies, particularly for infants and toddlers who may benefit from routine post-operative monitoring, lower thresholds for neurosurgical intervention, and closer provider-to-patient ratios to address their heightened postoperative vulnerability. Older children, who typically present with focal injuries and recover more favorably in the short term, require distinct preventive and management approaches. Public health efforts should also focus on fall prevention among toddlers—such as the installation of window guards—and road safety education for school-aged children. Moreover, the predominance of urban cases in our cohort and the underrepresentation of rural infants may reflect both survival bias and disparities in access to care. Prior studies have similarly reported worse trauma outcomes in rural-dwelling children, often linked to transport delays and limited recognition of serious injury [26]. Future research and health-system planning should therefore prioritize rural outreach, systematic data collection, and strengthened pediatric TBI recognition and response in peripheral centers.

This study presents an age-stratified analysis of neurosurgical TBI outcomes in a Bolivian pediatric population, providing novel insights from a low-resource Latin American context. Our 5-year dataset comprises a substantial number of surgically managed cases, featuring detailed metrics on operative timing, complications, and reintervention. Using WHO-defined developmental age categories allowed a nuanced interpretation of physiologic and injury pattern differences. Conducted at Bolivia’s national pediatric neurosurgical referral center, the study reflects real-world patient flows and resource limitations. Importantly, despite infrastructural constraints, our 97% in-hospital survival rate aligns with outcomes in high-income countries. For example, Lu et al. reported a 4.0% in-hospital mortality rate in a large U.S. pediatric TBI cohort [20]. The significance of these findings highlights the potential for high-quality neurosurgical care even in low-resource settings.

Several limitations must be acknowledged. This single-center study, while representative of Bolivia’s public neurosurgical sector, limits broader generalizability. Nearly 43% of survivors were lost to follow-up, restricting the assessment of long-term neurological and developmental outcomes. Prior work suggests that even when young children survive TBI, they are more likely to experience lasting cognitive deficits due to disrupted brain development [3, 26]. Additionally, system-level factors such as ICU availability, which likely contributed to surgical delays and complication rates, were not directly measured. The severity of injury could not be quantified, as Glasgow Coma Scale (GCS) or Abbreviated Injury Scale (AIS-Head) scores were not recorded in the hospital’s electronic registry, precluding risk-adjusted outcome comparisons across age groups. Although our research team prospectively collected this cohort and represents all surgically managed pediatric TBI cases at the country’s primary referral hospital, Bolivia lacks a centralized national TBI registry; thus, the dataset does not capture patients managed non-operatively or at hospitals without neurosurgical capacity. Consequently, these 165 cases likely represent the most comprehensive operative series currently available but do not encompass all pediatric TBI cases nationwide. Future prospective, multicenter collaborations and the establishment of a national trauma registry would help validate and expand upon these findings.

## Conclusion

Pediatric traumatic brain injury (TBI) poses a significant burden in low-resource settings, where age-related physiological differences demand tailored care strategies. In this Bolivian neurosurgical cohort, infants and toddlers experienced disproportionately high complication and reoperation rates, highlighting their vulnerability despite comparable survival to older children. These findings underscore the importance of age-adapted protocols that prioritize timely surgical intervention and vigilant post-operative care for the youngest patients. Simultaneously, age-specific prevention efforts (such as fall prevention in toddlers and traffic safety education for school-aged children) remain essential. Addressing these age-dependent differences in both treatment and prevention is key to improving outcomes across the pediatric TBI spectrum, particularly in resource-limited environments.

## Data Availability

The datasets generated and/or analyzed during the current study are available from the corresponding author upon reasonable request, subject to institutional ethical guidelines and approval.

## References

[1] Araki T, Yokota H, Morita A (2017) Pediatric traumatic brain injury: characteristic features, diagnosis, and management. Neurol Med Chir (Tokyo) 57(2):82–93. 10.2176/nmc.ra.2016-0191 28111406 10.2176/nmc.ra.2016-0191 PMC5341344

[2] Purcell LN et al (2020) Survival and functional outcomes at discharge after traumatic brain injury in children versus adults in resource-poor setting. World Neurosurg 137:e597–e602. 10.1016/j.wneu.2020.02.062 32084614 10.1016/j.wneu.2020.02.062 PMC7202968

[3] Nacoti M et al (2020) Addressing key clinical care and clinical research needs in severe pediatric traumatic brain injury: perspectives from a focused international conference. Front Pediatr 8:594425. 10.3389/fped.2020.594425 33537259 10.3389/fped.2020.594425 PMC7849211

[4] Kennedy L et al (2022) Moderate and severe TBI in children and adolescents: the effects of age, sex, and injury severity on patient outcome 6 months after injury. Front Neurol 13:741717. 10.3389/fneur.2022.741717 35989939 10.3389/fneur.2022.741717 PMC9382186

[5] Figaji AA (2017) Anatomical and physiological differences between children and adults relevant to traumatic brain injury and the implications for clinical assessment and care. Front Neurol 8:685. 10.3389/fneur.2017.00685 29312119 10.3389/fneur.2017.00685 PMC5735372

[6] Sariaslan A et al (2016) Long-term outcomes associated with traumatic brain injury in childhood and adolescence: a nationwide Swedish cohort study of a wide range of medical and social outcomes. PLoS Med 13(8):e1002103. 10.1371/journal.pmed.1002103 27552147 10.1371/journal.pmed.1002103 PMC4995002

[7] Taylor HG et al (2008) Traumatic brain injury in young children: postacute effects on cognitive and school readiness skills. J Int Neuropsychol Soc 14(5):734–745. 10.1017/S1355617708081150 18764969 10.1017/S1355617708081150 PMC2733858

[8] Beauchamp M et al (2024) Early childhood concussion. Pediatrics. 10.1542/peds.2023-065484 39380506 10.1542/peds.2023-065484

[9] Liang KWH et al (2024) Differences in clinical outcomes and resource utilization in pediatric traumatic brain injury between countries of different sociodemographic indices. J Neurosurg Pediatr 33(5):461–468. 10.3171/2024.1.PEDS23306 38364231 10.3171/2024.1.PEDS23306

[10] Haarbauer-Krupa J et al (2021) Epidemiology of chronic effects of traumatic brain injury. J Neurotrauma 38(23):3235–3247. 10.1089/neu.2021.0062 33947273 10.1089/neu.2021.0062 PMC9122127

[11] Woo J et al (2025) Management and outcomes of pediatric traumatic brain injury in Nigeria: a systematic review. J Neurosurg Pediatr 35(6):627–636. 10.3171/2024.12.PEDS24437 40184688 10.3171/2024.12.PEDS24437

[12] Lu V et al (2024) Assessing pediatric neurosurgery capacity in La Paz, Bolivia: an illustrative institutional experience of a lower-middle-income country in South America. J Neurosurg Pediatr 34:1–9. 10.3171/2024.3.PEDS24126 38788242 10.3171/2024.3.PEDS24126

[13] Mediratta S et al (2021) Barriers to neurotrauma care in low- to middle-income countries: an international survey of neurotrauma providers. J Neurosurg 137(3):789–798. 10.3171/2021.9.JNS21916 34952519 10.3171/2021.9.JNS21916

[14] Shrestha G, Goffi A, Aryal D (2016) Delivering neurocritical care in resource-challenged environments. Curr Opin Crit Care 22:1. 10.1097/MCC.0000000000000285 26828423 10.1097/MCC.0000000000000285

[15] Hwang SK, Kim SL (2000) Infantile head injury, with special reference to the development of chronic subdural hematoma. Childs Nerv Syst 16(9):590–594. 10.1007/s003810000312 11048634 10.1007/s003810000312

[16] World Health Organization. Guidelines for essential trauma care. Geneva: WHO; 2012. Available: https://www.who.int/publications/i/item/guidelines-for-essential-trauma-care

[17] Cohen Hubal EA et al (2014) Identifying important life stages for monitoring and assessing risks from exposures to environmental contaminants: results of a World Health Organization review. Regul Toxicol Pharmacol 69(1):113–124. 10.1016/j.yrtph.2013.09.008 24099754 10.1016/j.yrtph.2013.09.008 PMC5355211

[18] Graves JM et al (2025) Pediatric severe TBI in South America: healthcare resource utilization before and during the COVID-19 pandemic. PLoS Glob Public Health 5(5):e0004318. 10.1371/journal.pgph.0004318 40338920 10.1371/journal.pgph.0004318 PMC12061101

[19] Joyce T, Gossman W, Huecker MR. Pediatric abusive head trauma. In: StatPearls. Treasure Island (FL): StatPearls Publishing; 2025. 29763011

[20] Lu V, Hernandez N, Wang S (2022) National characteristics, etiology, and inpatient outcomes of pediatric traumatic brain injury: a KID study. Childs Nerv Syst. 10.1007/s00381-022-05544-1 35499615 10.1007/s00381-022-05544-1

[21] Barthélemy EJ et al (2019) Injury-to-admission delay beyond 4 hours is associated with worsening outcomes for traumatic brain injury in Cambodia. World Neurosurg 126:e232–e240. 10.1016/j.wneu.2019.02.019 30825623 10.1016/j.wneu.2019.02.019

[22] Gupta S, et al. Third delay in traumatic brain injury: time to assessment as a vital predictor of mortality. Neurosurgery. 2018;65. Available: https://journals.lww.com/neurosurgery/fulltext/2018/09001/188_third_delay_in_traumatic_brain_injury__time_to.109.aspx 10.3171/2018.8.JNS182182 30660121

[23] Grevfors N et al (2020) Delayed neurosurgical intervention in traumatic brain injury patients referred from primary hospitals is not associated with an unfavorable outcome. Front Neurol 11:610192. 10.3389/fneur.2020.610192 33519689 10.3389/fneur.2020.610192 PMC7839281

[24] Xue S, Zhang Z, Liu Y (2025) Effects of intracranial pressure monitoring in pediatric severe traumatic brain injury: a meta-analysis of cohort studies. Front Neurol 16:1557820. 10.3389/fneur.2025.1557820 40166641 10.3389/fneur.2025.1557820 PMC11955484

[25] Ajoku U, Hawryluk G, Kullmann M (2024) Intracranial pressure monitoring and treatment practices in severe traumatic brain injury between low-and middle-income countries and high-income countries: data or dogma? Surg Neurol Int 15:368. 10.25259/SNI_251_2024 39524597 10.25259/SNI_251_2024 PMC11544475

[26] Patel PD et al (2021) Measuring the effects of institutional pediatric traumatic brain injury volume on outcomes for rural-dwelling children. J Neurosurg Pediatr 28(6):638–646. 10.3171/2021.7.PEDS21159 34598145 10.3171/2021.7.PEDS21159

